# Construction of a new LED chamber to measure net ecosystem exchange in low vegetation and validation study in grain crops

**DOI:** 10.1038/s41598-023-39148-9

**Published:** 2023-07-22

**Authors:** Taewhan Shin, Wei Xue, Jonghan Ko

**Affiliations:** 1 Applied Plant Science, Chonnam National University, 77 Yongbong-ro, Buk-gu, Gwangju, 61186 South Korea; 2 State Key Laboratory of Grassland Agroecosystems, College of Ecology, Lanzhou University, Lanzhou, 730000 China

**Keywords:** Agroecology, Ecosystem ecology

## Abstract

A vegetation canopy chamber system measures gas exchanges in the field between plants and the environment. Transparent closed chambers have generally been used to measure canopy fluxes in the field, depending on solar radiation as the light source for photosynthesis. However, measuring canopy fluxes in nature can be challenging due to fluctuations in solar radiation. Therefore, we constructed a novel transient-state closed-chamber system using light-emitting diodes (LEDs) as a light source to measure canopy-scale fluxes. The water-cooled chamber system used a 1600 Watt LED module to produce constant photosynthetically active radiation (PAR) and a CO_2_ gas analyzer for concentration measurements. We used the LED chamber system to measure barley and wheat gas exchanges in the field to quantify CO_2_ fluxes along a PAR gradient. This novel technology enables the determination of photosynthesis rates for various crops under diverse environmental conditions, in diverse ecosystems, and across long-term interannual changes, including those due to climate change.

## Introduction

Terrestrial carbon fluxes within the plant-atmosphere continuum play an essential role in the ecosystem carbon cycle^1,2^. For instance, crop plant CO_2_ uptake through stomata is expressed as gross primary production (GPP) or as the apparent photosynthetic integration^3^. In addition, the crop canopy releases CO_2_ into the atmosphere through plant respiration, which includes the processes of dark respiration and photorespiration. Therefore, the assessment of CO_2_ fluxes in an agricultural ecosystem is critical for explaining the physiological responses of crops to environmental variables. Furthermore, enhanced insight into these ecosystem processes will aid in the projection of food crop production under climate change models and in the estimation of carbon sequestration in the soils by atmospheric CO_2_ fixation^4,5^. Several techniques are available to measure crop canopy CO_2_ fluxes, namely, the tower system using eddy covariance (EC) and gradient methods, the steady-state chamber system, and the transient-state closed-chamber system^6–11^.

An EC tower system is commonly used to monitor atmospheric gases (e.g., CO_2_ and methane), water vapor, and heat fluxes above plant canopies using micrometeorological methods^12–14^. It has the advantage of not disturbing the environment around the vegetation canopy and is effective for long-term and stationary monitoring of CO_2_ and water balances at land surfaces. The EC tower system requires a substantial open-field space (> 100 m) to achieve stable flux measurements^15,16^. However, this requirement is unreasonable for conventional agronomic plot experiments. Therefore, canopy-chamber methods (the steady-state open-system and the transient-state closed-system methods) remain the exclusive methodology for plot-sized agricultural experiments^17,18^. Steady-state open-systems, including open-top chambers, are most commonly used for long-term experiments of field crops exposed to elevated CO_2_ concentration^10^. However, these systems require climate control and flow measurements, while bearing the attendant disadvantage of modifying the microclimate of the crop canopies^17^. In contrast, transient-state closed-chamber systems can be operated with minimal alterations to microclimate conditions or flow measurement.

Transient-state closed-chamber systems are a practical option for plot-sized agricultural experiments^18^. These systems are employed to precisely measure CO_2_ and water vapor fluxes between the atmosphere and crop canopies^19–22^. The added advantages of the transient-state-type chamber systems are their portability and relatively low cost, deliverance of easily quantifiable outputs, and ease of operation by individuals^19^. In addition, these chamber systems facilitate sufficient experimental replication and cause only minor interruptions of the crop environment because they are positioned over plant canopies for a short time (about a couple of minutes) and then removed, ready for the next measurement^20^. However, they may be limited to only determining optimum plant CO_2_ fluxes due to solar radiation fluctuations during cloudy days within a period of a few days in specific environments, being more severely restricted in the case of intra-day measurements. This limitation can be especially severe in monsoon weather conditions over the crop-growing season. Since light-emitting diode (LED) has advantages over natural light in terms of uniformity and controllability for photosynthesis quantification studies, LED-based leaf-scale chamber systems have been commonly employed in field studies^23–25^. However, to the best of our knowledge, no attempt has been made to create an LED-based canopy-scale chamber system that controls light levels within its chamber. Therefore, to overcome this problem, we constructed a new portable transient-state LED closed-chamber system to measure the gas fluxes of plant canopies with extended applicability.

## Results

**Figure 1:**
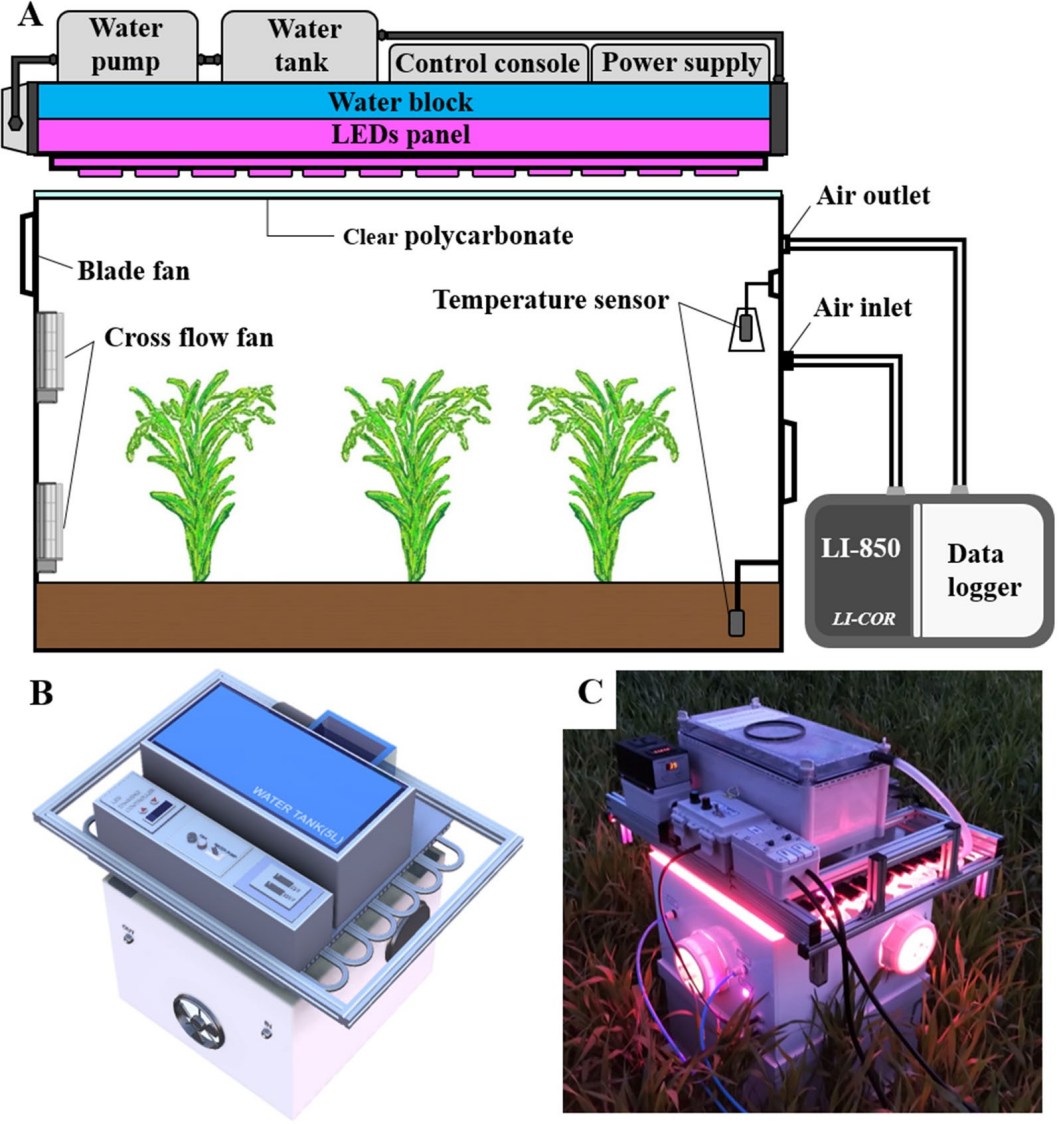
Construction of the light-emitting diode (LED) chamber system. (**A**) A schematic illustration of the LED chamber system used to determine the CO_2_ fluxes of plant canopies, composed of an LED panel block, a chamber body, and a data logger. (**B**) A pictorial representation of the system. (**C**) A photograph of an installed chamber system in a wheat field.

We constructed a transient-state pneumatic path closed-chamber system equipped with a synthetic illumination source using LEDs (Fig. 1A). The chamber system was composed of an LED module with a maximum power capacity of 1,600 W to control light levels, a water pump for cooling the LEDs, and a gas analyzer for flux measurement. The dimensions of the chamber module were 395 mm (W) × 395 mm (L) × 300 mm (H), enclosing up to four plant clusters with a crop planting density of 200 × 100 mm. This allowed for a plot-sized experimental vegetation canopy measurement. The LED system was designed to efficiently illuminate the interior and allow measurements of crop canopy gas fluxes with stable light conditions including the natural maximum, about 2,500 µmol m^−2^ s^−1^, and microclimate conditions. The LED chamber was designed to be capable of measuring net ecosystem exchange (NEE) and ecosystem respiration (R_eco_). If the power supply to the LED panel is interrupted, the LED chamber switches from acting as a transparent chamber to an opaque chamber, thus able to measure NEE as a transparent chamber and R_eco_ as a dark chamber. The photosynthetically active radiation (PAR) of LEDs can steadily increase up to 6,000 µmol m^−2^ s^−1^ at the maximum power capacity. In addition, the chamber system was designed for ease of operation in the field (Fig. 1B, C).

**Figure 2:**
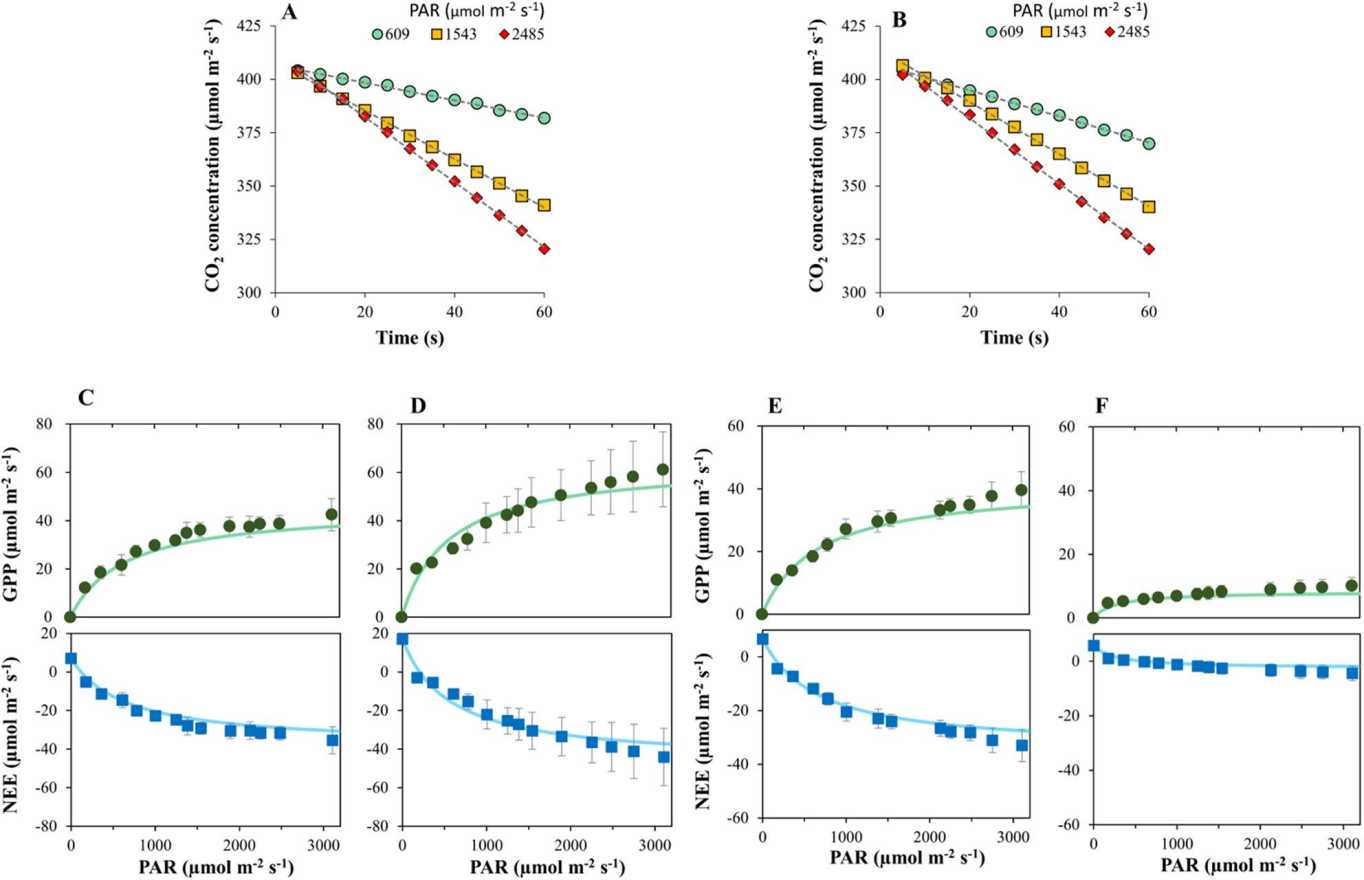
Measured CO2 fluxes using the LED chamber system. (**A**) Changes in the CO_2_ concentration inside the LED chamber system, measured at 5 s intervals, for wheat canopy at different intensities of photosynthetically active radiation (PAR). (**B**) Changes in the CO_2_ concentration inside the LED chamber system of barley at different intensities of PAR. Changes in gross primary production (GPP) and net ecosystem exchange (NEE) in response to PAR at (**C**) the maximum tillering stage and (**D**) the heading stage of barley, and at (**E**) the maximum tillering stage and (**F**) the heading stage of wheat.

To validate the efficacy of the chamber system, we measured the CO_2_ fluxes of two crops, barley and wheat, at two different growth stages to determine GPP and net ecosystem exchange (NEE) as a function of PAR (refer to Fig. S1). We confirmed that the CO_2_ concentration inside the LED chamber system continuously decreased while measuring barley and wheat canopies as the PAR intensity changed (Fig. 2A, B). The GPP and NEE responses of both crops exhibited conventional rectangular hyperbola curve shapes (Fig. 2C‒F). The GPP of barley at the maximum tillering and heading stages plateaued at 42.6 and 61.2 µmol m^−2^ s^−1^, while NEE stabilized at − 35.5 and − 44.1 µmol m^−2^ s^−1^, both at approximately 3,050 and 3,106 µmol m^−2^ s^−1^ of PAR, respectively (Fig. 2C, D; refer to Fig. S2). The GPP of wheat at the maximum tillering stage plateaued at 39.6 µmol m^−2^ s^−1^, while NEE dropped to − 33.0 µmol m^−2^ s^−1^, at approximately 3,106 µmol m^−2^ s^−1^ of PAR (Fig. 2E; refer to Fig. S2). The GPP and NEE of wheat at the grain-filling stage showed relatively reduced values and stabilized at 10.2 and − 4.5 µmol m^−2^ s^−1^ due to leaf senescence, leveling off at lower PAR values (Fig. 2F).

## Discussion

This study reports the novel formulation of a portable LED chamber system with a stable illumination source for the measurement of atmospheric gas fluxes in an agricultural ecosystem. While diverse closed-chamber systems have been developed to measure the exchange of atmospheric gases, including water vapor, in plant microenvironments^6,17–20,22,26–28^, to the best of our knowledge, no attempt has been made to create such an LED chamber system. Sophisticated chamber systems that are equipped with a light source comprise steady-state chamber systems^10,11,29^ or portable closed-chamber devices and are used for measuring leaf-level gas and water vapor exchanges^30,31^. The current LED chamber system has the advantages of portability and a stable light source and the capacity to quantify fluxes. This chamber is also suitable for characterizing radiation responses of plant canopies in less synthetic environments. While we cannot make direct comparisons of CO_2_ fluxes between natural light- and LED-based chamber systems for the same crops, it appears that the CO_2_ fluxes in the LED-based chamber are considered saturated at higher values than those in the natural light-based measurement counterparts, taking a look at the earlier reports by Choi et al.^22^ and Linder et al.^20,32^.

The LED chamber system’s weakness is that it is less portable, i.e., heavier than the alternative closed-chamber systems because of the inclusion of an additional LED and a water-based cooling structure. However, it is still operable by a single individual in the field. In addition, the new design holds similar sensitivity to the equivalent natural light-dependent closed chamber systems. For example, while measuring the gradients of gas fluxes between the chamber atmosphere and the environment outside the chamber, the temperature can be reduced due to forced air circulation^33^. We made ventilation holes in the chamber sides, which were sealed using closable caps during the measurement, to allow faster atmospheric air interchange in the current system (see Fig. 1C). Ventilation was performed using fans. In addition, the orientation of the leaves at the chamber boundaries is likely altered during positioning^26^. We assume that these problems do not ultimately decrease the measurement accuracy^27,28,33^ as they do not in the closed chamber systems mentioned earlier. A more challenging hamper for the current design is measuring H_2_O fluxes as a function of irradiance. Water vapor concentration increases rapidly, and the atmosphere within the chamber becomes fully saturated at approximately 2,000 µmol m^−2^ s^−1^ of PAR during measurement (heading stage). At the maximum tillering stage of barley and wheat, no condensation was observed even after a 2-min measurement, while condensation occurred after 90 s at the heading stage. Therefore, we limited the measurement time to 45 s in all growing seasons. We also found moisture condensation on the surface of the ice packs used to lower the temperature inside the chamber, resulting in a decrease in excess moisture. Although we could see a significant increase in the water vapor ratio inside the chamber during the measurements, we confirmed that it did not affect the estimation of NEE using the method reported by Rui^34^. We believe that this problem can be solved by making the chamber size larger to allow more room for air circulation. Another option would be to adopt an advanced air-cooling approach to eliminate moisture evaporation, e.g., using a micro air-conditioning system.

Further enhancements of the current LED chamber system could include advanced measurement capacities to allow more reliable determination of water vapor exchange, more sophisticated gas flux measurements, and automatic measurement capabilities^19,29^. Other added measurement capacities comprise enhanced measurement features, such as the stomatal conductance formulated in closed leaf chamber systems^30^, which would increase the applicability of the LED chamber system. Automating the LED chamber measurement process would help to enhance its performance precision and usability under field conditions. While it is out of the current research scope, additional studies would be needed to determine the characteristics of a consistent canopy flux for the typical growth stages of the crops of interest.

## Conclusion

The study showcases the development and validation of a newly designed LED chamber to measure net ecosystem exchange in low vegetation, focusing on grain crops like barley and wheat. This innovative LED chamber system effectively addresses the challenges associated with measuring canopy fluxes in nature, which are typically influenced by fluctuations in solar radiation. The system successfully measured gas exchanges in barley and wheat, quantifying CO_2_ fluxes along photosynthetically active radiation (PAR) gradient. The design of the LED chamber system enables accurate measurement of photosynthesis rates for various crops under diverse environmental conditions, including those induced by climate change. However, the system does have some limitations, such as its weight and the potential for rapid increases in water vapor concentration at high PAR levels. Nevertheless, this system represents a significant advancement in agricultural science. Future enhancements could involve more sophisticated gas flux measurements, advanced measurement capacities, and automatic measurement capabilities. This innovative technology substantially contributes to the field, especially given the urgent need for accurate, reliable measurements of gas exchanges in low vegetation and grain crops amidst climate change.

## Materials and methods

### Chamber construction

**Figure 3:**
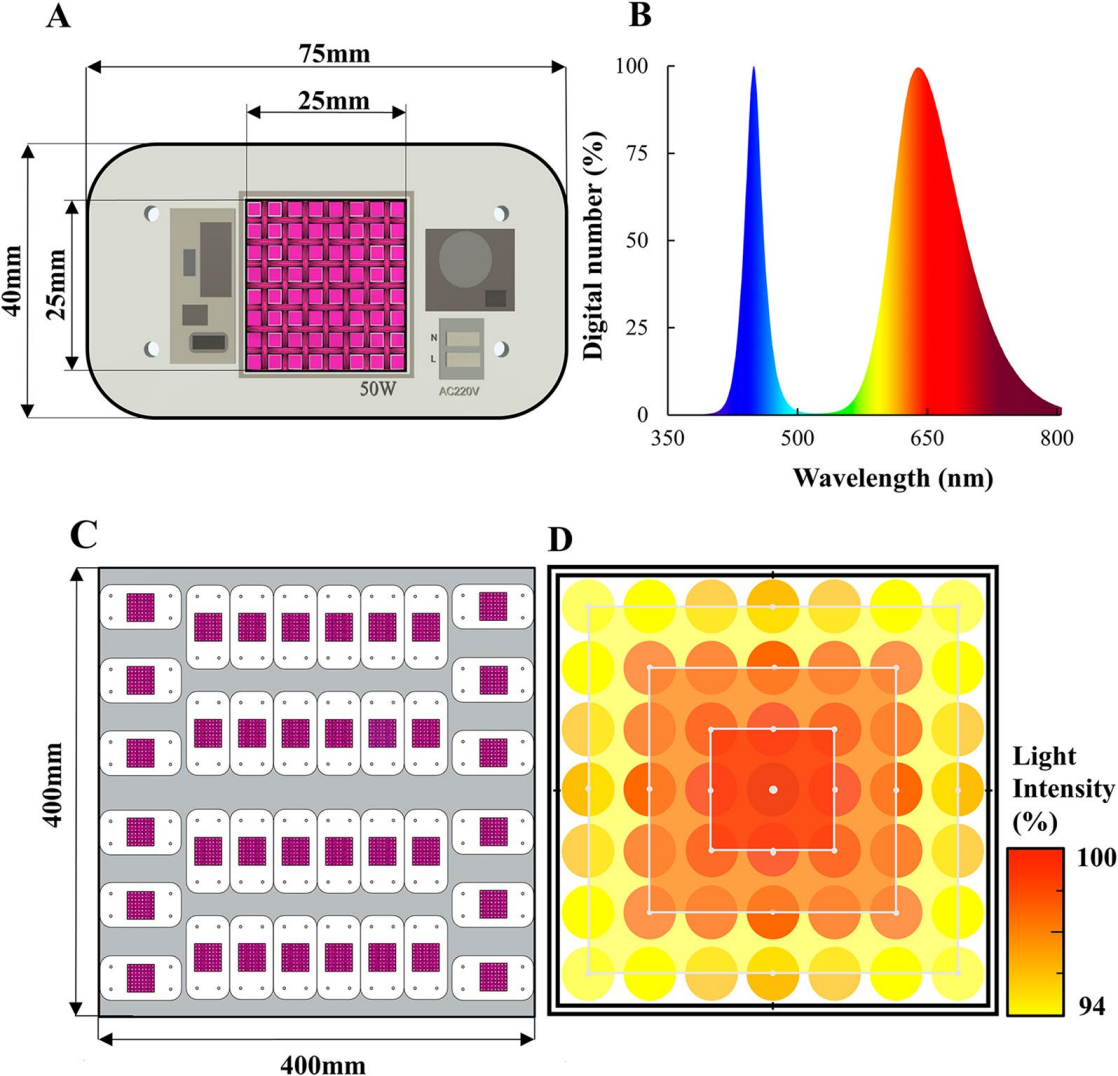
Characteristics of the light-emitting diodes (LEDs). (**A**) A pictorial representation of the LEDs used for the chamber fabrication. (**B**) Spectral response curves of the blue (center band, CB = 450 nm) and red (CB = 660 nm) LEDs with the absorption spectrum wavelength bands of chlorophyll a and b. (**C**) A pictorial representation of the constructed LED panel. (**D**) The hypothetical light intensity distribution.

A transient-state closed chamber was constructed (Fig. 1), comprising an LED panel, a chamber body, and a portable battery-operated infrared gas analyzer LI-850 (LI-COR, Inc., Lincoln, NE, USA). The LED panel was developed using high-brightness pcH-LEDs (Shenzhen Yuxinou Technology, Inc., Guangdong, China) with a spectrum close to the central wavelengths of chlorophyll a and b (Chlorophyll a: 430 and 660 nm, Chlorophyll b: 450 and 645 nm) to achieve photosynthetic activation (Fig. 3A, B). In addition, the photon flux in the far-red wavelength (701–750 nm) interacts more with shorter wavelength photons, enhancing photosynthetic activity in leaves under sunlight^35^. Of the total PAR of the pcH-LEDs, 11% is in the far-red spectrum (Fig. 3B). Therefore, the pcH-LED panel in the chamber enables users to create a light environment similar to the natural light environment in terms of photosynthetic activation. The 36 pcH-LED modules were assembled on a 400 × 400 × 3 mm aluminum board to maximize the uniformity of the light intensity distribution (Fig. 3C). In addition, a rectangular wall, 50 mm in height, covered with reflective inner sheets, was placed on the LED board edges to prevent light from exiting the chamber body and to ensure that the light from the LED module was uniformly mixed. As a result, we obtained a < 10% variation in the light intensity emanating from the panel (Fig. 3D). The light intensity of the LED panel was measured and calibrated using an LI-190SA quantum sensor (LI-COR, Inc.), and the wavelength spectrum within the chamber was acquired using an ASD FieldSpec3 spectroradiometer (Analytical Spectral Device, Inc., Boulder, CO, USA). The light intensity and uniformity measurements were obtained at a distance of 300 mm from the LED panel. The brightness inside the chamber was controlled from 0 to a maximum of 6,000 µmol photons m^−2^ s^−1^ using an LED panel console. Thus, the LED panel supplied various PAR intensities through the fine adjustment of the LED power supply (Fig. S3). The LED panel consumes up to 1,600 W at 220 V AC, supplied in our field experiment by a portable Honda EU30i generator with a 2.6 kW capacity (Honda Motor Co., Ltd., Tokyo, Japan). The other electric modules, including ventilation fans, circulation fans, and a water pump, were battery-powered at 12 V DC.

**Figure 4:**
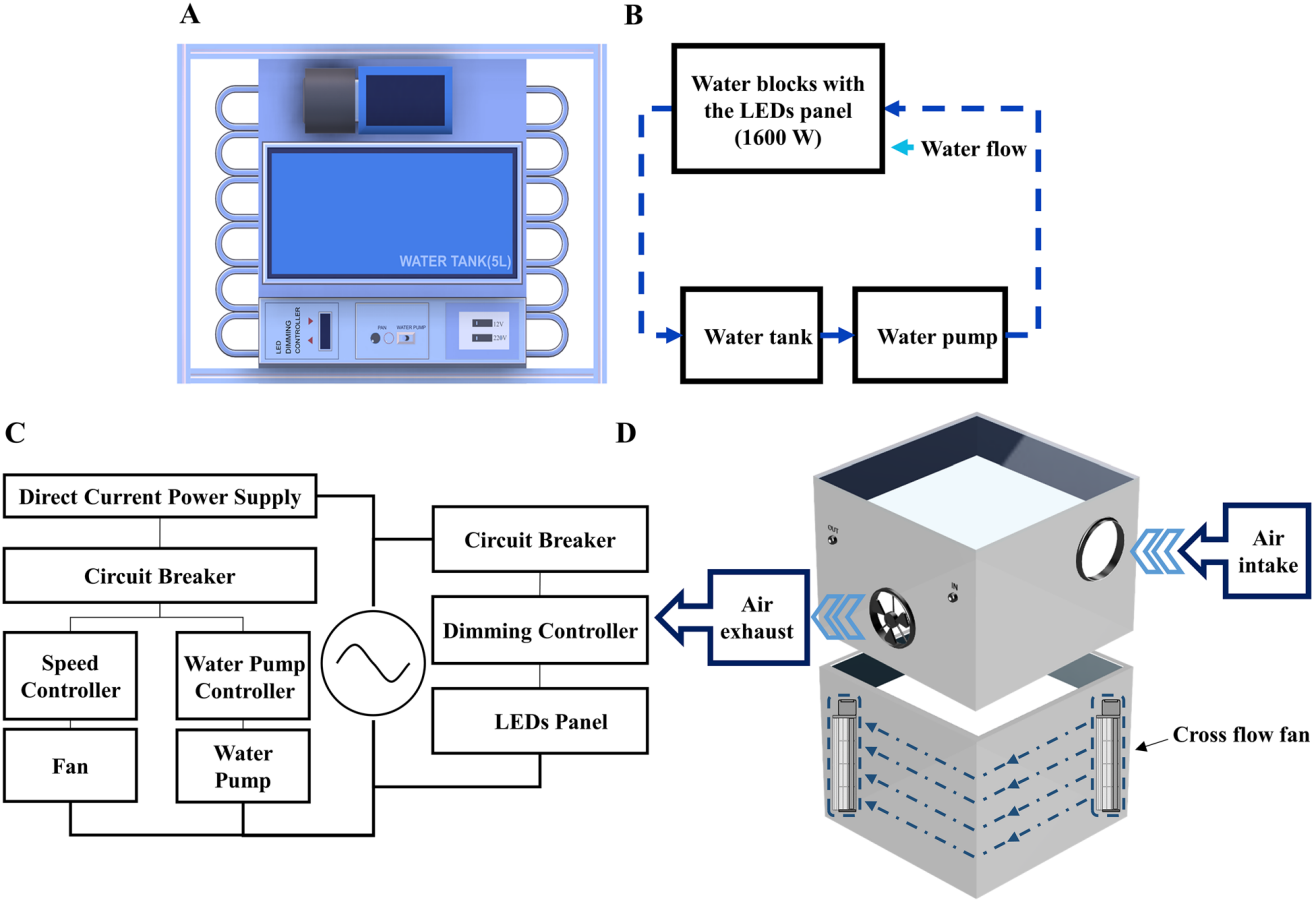
Features of the LED chamber system. (**A**) A pictorial representation of the water-cooling structure of the LED module, consisting of a water pump, water blocks, and a water tank. (**B**) The water circuit diagram of the water-cooling system. (**C**) An electric circuit diagram of the LED and water-cooling module systems. (**D**) A pictorial representation of the semi-automatic ventilation structure adopted for the chamber.

The high luminance LED panel not only provided variable light intensities but also generated varying levels of heat, which increased the temperature inside the chamber. Therefore, we developed a water-cooling system to minimize the temperature rise inside the chamber and light intensity changes caused by the heat load of the light source. The water-cooling system consisted of a water pump, a water block panel, and a water tank (Fig. 4A, B). The LED panel was placed on the water block panel. The coolant in the water tank circulated through the water block to cool the LED panel, thereby preventing a significant increase in temperature inside the chamber. A circuit diagram of the LED and water-cooling module electrical systems is presented in Fig. 4C.

The chamber body dimensions were 395 × 395 × 300 mm (46.8 L). Chamber extensions (250 and 500 mm in height) were also constructed to adjust the chamber height, depending on the crop canopies of interest. We employed white acrylic chamber walls because their high light reflection rate improved the light intensity within the chamber. The top panel of the chamber was made of a transparent polycarbonate board to allow the transmission of PAR from the LED panel. Each chamber was equipped with a ventilation system consisting of a pair of small cross-flow fans in opposite corners of the chamber walls to circulate the air and mitigate air temperature increases (Fig. 4D). Closable ventilation openings on both sides of the chamber body were designed to provide a rapid exchange between the atmosphere and the chamber using semi-automatic motor fans.

### CO_2_ flux measurement

Measurements of the CO_2_ flux of barley (*Hordeum vulgare*) and wheat (*Triticum aestivum*) canopies were obtained from the experimental research field at Chonnam National University (35°10′ N, 126°53′ E; 33 m), Gwangju, South Korea in 2020. The experimental research and field studies on these plants, including the collection of plant material, complied with relevant institutional, national, and international guidelines and legislation. Using the soil classification system of the National Institute of Agricultural Sciences (http://soil.rda.go.kr/eng/, accessed on February 8, 2022), the topmost layer (0‒30 cm) of the soil at the field site was categorized as loam (43.1% sand, 30.9% silt, and 26.0% clay). The soil had a pH (H_2_O) of 7.3; an organic carbon content of 17.0 g C kg^−1^; an available phosphorus (P) content of 535 mg P_2_O_5_ kg^−1^; a cation exchange capacity of 14.4 cmolc kg^−1^ (Ca^2+^ of 13.7, K^+^ of 0.3, and Mg^2+^ of 8.0); and a total nitrogen (TN) content, before fertilization, of 1.0 g N kg^−1^. The barley cultivar HeenChal and wheat cultivar Chokyung were sown on November 7, 2019, in a roughly 634.0 m^2^ area and harvested on June 20, 2020. The HeenChal barley and the Chokyung wheat were bred in 1993 and 2004, respectively, both at the National Institute of Crop Sciences, where voucher specimens of these materials have been deposited in a publicly available herbarium. Barley and wheat grain kernels were seeded using a mechanical seed drilling machine with a 0.2 m row spacings and a 0.1 m plant-to-plant spacings. The experimental blocks were arranged in a randomized complete block design with four replications. We installed two collar frames on each experimental block on which to place the LED chamber system, which measured NEE in the planted location and soil respiration in a bare soil location. Frames were installed on the soil surface to exclude the soil from the chamber volume^20,36^. Four grass clusters per frame were planted to measure CO_2_ flux in four frames in each plot. N, P, and K fertilizers were applied at 80, 70, and 35 kg ha^−1^, respectively. The N fertilizer was applied in three does: spread under the soil surface before seeding at 40% as a basal dosage and treated as a side dressing in 30% doses at the tillering and panicle initiation stages. Total doses of P and K were spread under the soil surface as basal applications before seeding.

We measured net gas exchange in the barley and wheat canopies according to previously described procedures^20,37^ using the LED chamber system with sensors to measure environmental variables (Table S1). The CO_2_ flux was determined for principal growth stages on day of year (DOY) 100‒104 (maximum tillering) and 118‒121 (heading) for barley and DOY 105‒107 (maximum tillering) and 132‒134 (grain filling) for wheat in 2020. In addition, PAR (400‒700 nm wavelengths) was measured within the chamber body using an LI-190SA quantum sensor (LI-COR, Inc.). The PAR value was adjusted by manipulating the light intensity of the LED panel. The chamber was then closed, and gas concentrations were measured after a sufficient time. The gap between the chamber and the collar frames on the ground was hermetically sealed with a rubber tube placed at the bottom of the chamber edge to avoid gas leakage^19,20^. Because the microclimate inside the chamber changed rapidly after closure, the measurement periods were made as short as possible, limited to approximately 45 s. After completing each measurement, the ventilation system was activated using internal and external fans to sufficiently mix the air inside and outside the chamber. Air strength was adjusted using the control console to establish an efficient circulation stream to help perform steady CO_2_ flux measurements. In addition, an ice pack was used to control the air temperature inside the chamber. It also reduced the moisture accumulation inside the chamber since humidity condensed on the surface of the ice pack. The measurements were undertaken sequentially in a systematic rotation using the LED chamber system, which was manually operated under all weather conditions. A programmable data logger CR300 (Campbell Scientific, Logan, UT, USA) was used to record the data every 5 s during each measurement period.

After excluding the data from the dead-band period where the output was zero^20,38,39^, CO_2_ fluxes were determined based on a linear regression describing the CO_2_ concentration change within the chamber as a function of time over the measurement period. CO_2_ flux was estimated using the equation of Flessa et al.^40^.

1 $${F}_{{CO}_{2}}={\mathrm{k}}_{{CO}_{2}}(273\times {T}^{-1}) (V\times {A}^{-1})({\mathrm{dc}}\times {\mathrm{dt}}^{-1})$$

where ${F}_{{CO}_{2}}$ is the CO_2_ flux (mg CO_2_ L^−1^ h^−1^), ${\mathrm{k}}_{{CO}_{2}}$ is the gas constant at 273.15 K (0.536 µg C µL^−1^), *T* is the air temperature (°K) in the chamber at the measurement time, *V* is the chamber volume (*L*), *A* is the surface area of the chamber collar, and dc × dt^−1^ is the CO_2_ concentration change in the chamber environment over time (CO_2_ mL L^−1^ h^−1^).

Gross primary production (µmol CO_2_ m^−2^ s^−1^) represents the CO_2_ fixed by vegetation canopy photosynthesis^3,41^. Therefore, GPP was estimated using the equation of Adiku et al.^42^.

2 $${\mathrm{GPP}}={\mathrm{R}}_{eco}-{\mathrm{NEE}}$$

where R_*eco*_ (µmol CO_2_ m^−2^ s^−1^) is the ecosystem respiration value.

A radiation response curve of NEE (µmol CO_2_ m^−2^ s^−1^) was estimated using the non-rectangular hyperbolic response model, also recognized as the Michaelis–Menten model^37,39,43^:

3 $${\mathrm{NEE}}=-\frac{\alpha \cdot \beta \cdot Q}{\alpha \cdot Q+\beta }+\upgamma$$

where α is the canopy radiation utilization efficiency (µmol CO_2_ m^−2^ s^−1^ per µmol photon m^−2^ s^−1^) as derived from the initial slope of the radiation response curve, β is the maximum CO_2_ uptake rate of the vegetation canopy (µmol CO_2_ m^−2^ s^−1^), Q is the photosynthetic photon flux density (µmol photon m^−2^ s^−1^), and γ is the average ecosystem respiration value (µmol CO_2_ m^−2^ s^−1^) during the measurement time (refer to Table S2). The hyperbolic radiation response model was adopted using a nonlinear least-squares fit approach^37,43^ and was used to estimate the continuous response of the CO_2_ flux to radiation changes.

### Relevant institutional, national, and international guidelines and legislation

Permissions to use the experimental field and cultivars were obtained from appropriate governing bodies.

## Supplementary Information

Supplementary Information.

## Data Availability

All data are available in the main text or supplementary information.
