# Supplementary material for: Construction of a new LED chamber to measure net ecosystem exchange in low vegetation and validation study in grain crops
Source: Sci Rep. 2023 Jul 22;13:11850. doi: 10.1038/s41598-023-39148-9 (PMC10363127; doi:10.1038/s41598-023-39148-9)
Supplement: Supplementary file 1 — Supplementary Information. [file 41598_2023_39148_MOESM1_ESM.docx]

Supplementary Information for

Construction of a New LED Chamber to Measure Net Ecosystem Exchange in Low Vegetation and Validation Study in Grain Crops

Taewhan Shin, Wei Xue, Jonghan Ko*

*Corresponding author. Email: jonghan.ko@jnu.ac.kr


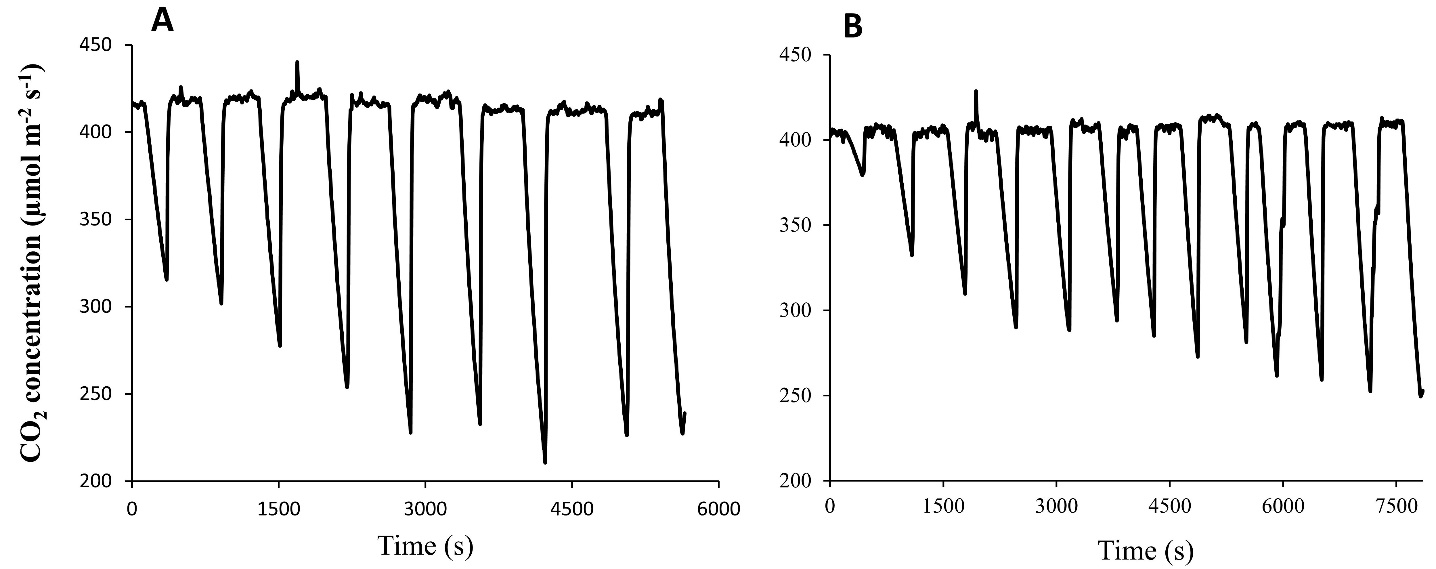


**Fig. S1.** Changes in CO_2_ concentration in (A) barley and (B) wheat canopies inside the chamber at 5-second intervals in the field. The rate of CO_2_ uptake by the canopy was calculated based on determining the gradient of changes in CO_2_ concentration over time after the cover was closed.


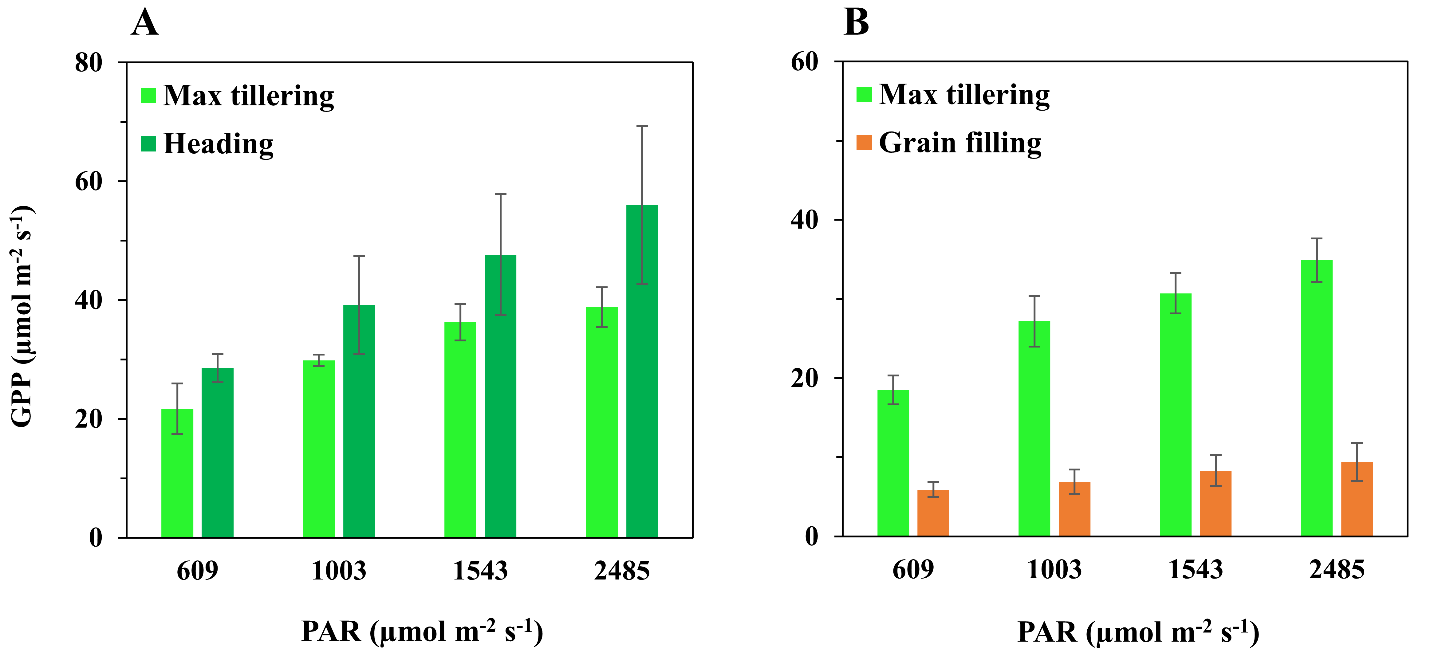


Fig. S2. Changes in gross primary production (GPP) with respect to distinct ranges of photosynthetically active radiation (PAR). (A) GPP changes of barley at the max tillering and heading stages. (B) GPP changes of wheat at the max tillering and grain filling stages.


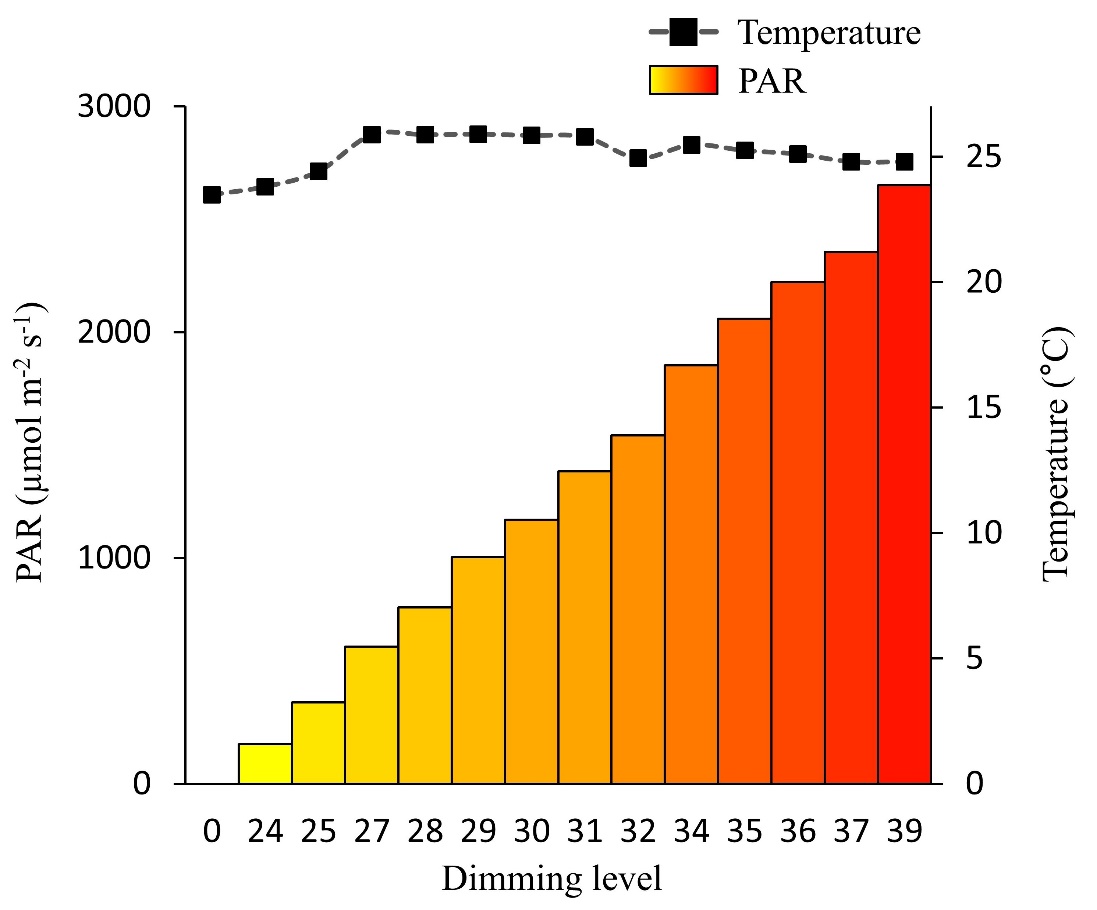


**Fig. S3.** PAR and temperature changes in the LED chamber in response to the control console operation.

Table S1. Sensor information used to measure environmental variables in the light-emitting diode chamber system.

| Variable | Sensor | Input power  (V) | Output power  (V) | Measuring range |
| --- | --- | --- | --- | --- |
| CO_2_ | Infrared gas analyzer^†^ | 100 to 240 AC | 12 DC | 0-20000 ppm |
| Air temperature | Thermometer A^‡^ | 9 DC | N/A | −100 to 1300 ℃ |
| Soil temperature | Thermometer B^§^ | 9 DC | N/A | −50 to 1300 ℃ |
| PAR^*^ | Quantum sensor^¶^ | N/A | N/A | 0-3000 µmol |

^*^ PAR stands for photosynthetically active radiation. †LI-850 (LI-Cor, Inc., Lincoln, NE, USA). ‡TM-926 (Lutron Electronics, Inc., Coopersburg, PA, USA). §TM-902C (Lutron Electronics, Inc., Coopersburg, PA, USA). ¶LI-190R (LI-Cor, Inc.).

Table S2. CO_2_ flux parameters used to calculate net ecosystem exchanges of barley and wheat growth stages.

| Parameter | Barley | |  | Wheat | |
| --- | --- | --- | --- | --- | --- |
|  | Max tillering | Heading |  | Max tillering | Grain filling |
| α^*^ | 0.07 | 0.11 |  | 0.06 | 0.03 |
| *β*^†^ | 45.38 | 64.0 |  | 41.44 | 8.26 |
| γ^‡^ | 7.08 | 17.11 |  | 6.65 | 5.70 |

^*^ (µmol CO_2_ m^−2^ s^−1^ per µmol photon m^−2^ s^−1^) is the canopy radiation utilization efficiency; ^†^*β* (µmol CO_2_ m^−2^ s^−1^) is the maximum CO_2_ uptake rate of the plant canopy; ^‡^γ (µmol CO_2_ m^−2^ s^−1^) is the average ecosystem respiration.
